# Inhibitory STDP generates inverse models through detailed balance

**DOI:** 10.1186/1471-2202-14-S1-O3

**Published:** 2013-07-08

**Authors:** Maren Westkott, Christian Albers, Klaus Pawelzik

**Affiliations:** 1 Institute for Theoretical Physics, University of Bremen, 28359 Bremen, Germany

## 

In the song bird ('backward') mappings from sensory representations to motor areas recently were proposed that would 'postdict' the motor activations during singing. Such a sensor-motor mapping represents an inverse model of the motor-sensor-loop passing through the world and thereby can explain the impressive imitation capabilities of song birds [1].

The neurobiological mechanisms that might generate, fine tune and continuously adapt such inverse models, however, are not known. Here we show that spike timing dependent plasticity (STDP) of the inhibitory synapses is sufficient for the self-organisation of the inverse model in a simple closed loop motor-sensor-motor system [2].

Similar to the case of forward models, where predictable, self-generated inputs become suppressed [3], the proposed mechanism generates sparse motor activities by cancelling predictable fluctuations of the neurons' excitabilities. Our results show that inverse mappings can be learned with an elementary and biologically plausible learning rule and thus could underly imitation learning. In our presentation we will discuss also the potential relevance of this mechanism for the operation state of recurrent networks as e.g. cortex [4].
